# Letter from Prof. White

**Published:** 1851-11

**Authors:** 


### EDITORIAL DEPARTMENT.

*Letter from Prof. White.* — The following letter, which has been received just as we are called on to supply copy for this department of our Journal, is inserted in lieu of editorial matter. The succedaneum will doubtless be acceptable to our readers. Prof. W. was to have sailed in the steamer Franklin, from Havre, on the 22d ult., and may be expected to arrive soon after the first of the present month.— EDITOR.

VIENNA, August, 1851.

*My Dear Doctor,* — I now write you from the "gay" capital of the Austrian empire. On my way from Berlin I took Prague in my route for the purpose of visiting its University, one of the oldest and most renowned institutions in Europe. I desired also particularly to see the present professor of obstetrics in the medical department of that institution, Dr. Kevish. Prof. Kevish is conceded to be, at this moment, the leading mind in Germany in his department. Judging from his contributions to science, he must possess an inventive genius, and is beyond all question a very industrious student and most acceptable teacher. I was not fortunate enough to find him at home, and I regret to say he was absent in consequence of ill-health, and that apprehensions are entertained by his friends that he may never recover.

I found this venerable medical institution still maintaining a good reputation, with a large class, an able faculty, and every facility for instruction which a good museum, with ample apparatus, could supply. But the increasing importance attached, in modern times, to clinical instructions, in comparison with the now nearly obsolete theoretical course pursued almost exclusively by the ancients, will eventually compel it to succumb to the schools attached to the large hospitals of the capital. This University was the first great public school founded in Germany. The fame of the teachers, and the privileges granted to scholars, was such at one time, as to attract hither students from all parts of Europe. Some idea of its importance at that time, may be formed by mentioning the fact that in 1409, in consequence of a measure proposed by John Huss for abridging the privileges of the foreigners and transferring the preponderance from them to the Bohemians, there seceded in one week not less than 25,000 students. This seems almost incredible, but the entire body of students is estimated by writers of that period at 40,000. The movement resulted in the more general diffusion of science, as the seceders dispersed themselves over Europe, and became the founders of the Universities of Liepzig, Heidelburg, Cracow, &c.

The traveler is greatly annoyed, both at Prague and Vienna, at this time, by the passport regulations. These cities are in a "state of siege," as it is termed, being, in consequence of the recent revolutionary troubles, placed under the control of the minister of war, martial law taking the place of civil. The catechising relative to place of birth, age, whether married or unmarried, business, places of departure and destination, &c., &c., are exceedingly minute and vexatious. Indeed, it has been jocosely remarked that for biographical statistics relative to any one who may have traveled in Austria during the last two years, you have but to refer to the records of the police, where the fullest information can be obtained. Notwithstanding all these embarrassments, the medical observer is richly repaid for his trouble by a visit at the large and well conducted hospitals under the direction of men second to none in point of industry or acquirement. It may be mentioned in confirmation of the latter remark, that not only do most of these men write and speak English, French, and the other modern languages, with facility, but very many are able to converse or lecture in the *Latin* with ease and fluency. It is equally certain that in every department of medicine they are thoroughly posted, and actively engaged in new investigations. In pathology they are second only to the Parisians, and in chemistry, animal and vegetable, the Germans have placed themselves in advance of all the other European schools by their recent researches and discoveries. In clinical teaching the hospitals of Vienna afford a most extensive field which is fully improved in every department. As a whole, so far as limited opportunities would warrant an opinion, the bed-side remarks are more full and practical than in the Parisian hospitals. Much more attention is paid to the *treatment* of the various diseases submitted to the observation of the pupil, though it can scarcely be possible to exceed the French in description of symptoms and accuracy of diagnosis.

The *General Hospital* in Vienna, is an immense establishment, covering several acres with its different wards and courts. It is probably the largest hospital in the world, having about four thousand beds, and receiving annually more than 300,000 patients. It has ample grounds for the convalescent to exercise in the air, in well shaded parks cooled by numerous fountains, which are constantly sending forth an abundant supply of pure water. These buildings, or rather this city of the sick, has a front of about twelve hundred feet, and possesses considerable architectural beauty. The wards, one hundred and eleven in number, are high and well ventilated, and the most perfect cleanliness is observed throughout. The nurses are hired, and, although all necessary attention is doubtless bestowed, they seemed less considerate of the comfort of the patient than those self-devoted females who, without any other remuneration than the consciousness of relieving suffering humanity, and the hope of a future reward, render the same services in most catholic countries.

The accommodations for lying-in women, are upon the same magnificent plan as the other departments. There are not less than eight thousand females annually delivered in this institution. The number of labors is seldom less than fifteen or twenty in the twenty-four hours, and sometimes amounts to thirty or forty. It is, as you perceive, therefore, the most extensive institution of the kind in the world, and affords the most ample opportunity for clinical instruction in this important department. Professor Klein and Dr. Braun, have the supervision of the lying-in wards at this time, and several hours are given daily to the instruction of their pupils. It rarely happens that there are not one or more cases of labor in the *salle d'accouchment*, giving unparalleled opportunities to the young obstetrician for experience and observation. The result, as you would anticipate, is to induce such students, as can afford the pecuniary expense, and who wish to perfect themselves in this branch, to visit this city before commencing practice. At this moment there are pupils here from England, Ireland, and Scotland, and even from the southern and western American states. The institution is in great favor with the citizens; females consider themselves much safer here than elsewhere, and admission is therefore coveted. Among the things which first attracted my attention upon visiting this institution, was the care observed in preventing the propagation of any contagious disease, more especially to guard against the development or propagation of puerperal fever, which is the great scourge of most hospitals of this kind. No one is permitted to examine a female *per vaginam*, after making post mortem examinations, no matter with what disease the body examined was affected, until thorough ablution has been resorted to, some disinfecting solution applied to the hands, and the clothes changed. No one is permitted to make a vaginal examination of a second female without previous ablution. Again, the wards of the hospital for the sick, although so constructed as to pass from *salle* to *salle*, are ordinarily kept closed, being entered from without only, and preventing the passage of air from hall to hall, and the possibility of thus propagating disease. These precautions appear to me to possess value, and, so far as experience has been had, there seems a diminution in the proportion of mortality. It is true that all do not concede puerperal fever to be communicable, but there are many facts upon record which it would be difficult to explain in any other way, and until its noncontagiousness is incontrovertibly established, all humane practitioners will exercise the same caution which would be necessary to prevent its extension did they acknowledge it susceptible of being thus propagated.

Much discussion has been had, and many plans devised for exciting premature labor in those unhappy cases of deformity in which delivery at term is ascertained to be impossible. Within the last few months two new methods of dilating the uterine outlet, and exciting the expulsion of its contents have been resorted to. The idea of the application of a jet of water originated with Dr. Kevish, of Prague, if I mistake not. It consists in the forcible application or direction of a current of water upon the mouth of the womb at intervals until labor pains are excited. Dr. Braun showed me his apparatus for this purpose. It consists of a syringe, which will hold sixteen or eighteen ounces, with a flexible tube attached of such length as to penetrate to the upper portion of the vagina. This is filled with water, cold or warm, the latter being less likely to give pain at the time, the tube is then introduced into the vagina, and its contents forcibly thrown against the uterine outlet. If the syringe be double, possessing a valve, so that it may be refilled several times without withdrawing it, so much the better. This stream should be continued for four or five minutes, and then the apparatus is withdrawn. If the first injection be not followed by pains, it should be repeated in a few hours. The same apparatus, I may here add, by substituting a metallic tube with a bulbous extremity perforated much like the spout of a gardener's watering-pot, is used to administer a tonic or cold douche in cases of leucorrhœa or debility of these organs in the unimpregnated state. In eighteen cases in which this instrument has been resorted to in this hospital during the last few months, the average time before the supervention of labor after the first injection has been about four hours.

The second method, which originated with Dr. Braun, consists in inserting in the vagina an elastic bag or pouch, with a long neck and a stop cock attached at its extremity, then filling it with water, and gradually distending it. The same form of bag is often used in Paris and America as a tampon, being inflated after insertion. The liquid is thrown through the tube, by means of a syringe, as rapidly as the vagina will dilate without pain, and the stop cock turned after each injection. The injection may accordingly be repeated every hour until labor supervenes. Dr. Braun, its inventor, calls this apparatus the "vaginal extensor for procuring premature labor." He has used it in the 5th, 7th, 8th, and 9th months of pregnancy, and has never failed in bringing on labor in a few hours. As to the *modus operandi*, the most probable explanation seems to be that, by means of the jet of water in the one case, and by the complete distension of the vagina and consequent effect upon the os which forms the upper floor of this canal in the other, the membranes are detached from the uterine orifice which is itself dilated, and the uterus excited to contraction. Be the explanation what it may, it is certain that in those cases of eclampsia or pelvic contraction requiring the premature expulsion of the contents of the uterus, it would seem more simple to resort to either of the above methods than to any of the mechanical means for dilating the os heretofore in vogue. It is more readily practiced also than the puncturing of the membranes, possessing the additional advantage of leaving these tissues entire to retain the liquor amnii until a later period, increasing, thereby, the safety of both mother and child. It is of course presumed, in the preceding remarks, that the necessity for interference has been established beyond the possibility of doubt. The elastic bag would seem admirably adapted to fulfill the indications present often in cases of inevitable abortion, accompanied with hæmorrhage. The "extensor," when fully dilated, filling the vagina, will arrest the flow of blood, the mouth of the uterus will be opened, and the expulsion of the ovum secured.

I found in use, also, in this hospital, a new instrument for returning the funis within the uterine cavity, when prolapsed during labor, which deserves to be mentioned. Dr. Braun, its inventor, calls it the "repositorium." It has been used in thirty or forty cases of this unfortunate complication, during the last two years, and the record shows a degree of success which can be expected to follow no other plan of treatment with which I am acquainted. The instrument in question, is made of gutta percha, is about eighteen inches long, five-eighths of an inch in diameter at the larger extremity, and three-eighths at the smaller, the latter being smooth and rounded at the point. It has a small perforation about an inch from the smaller extremity, through which a loop of tape is passed. The free ends of the tape are long enough to extend to the base of the instrument, the prolapsed funis is enclosed in this loop of tape, and the latter then carried over the small end of the repositorium, whilst the free ends at the base are tightened and secured by bein several times passed round it. This encloses the funis simply between the loop and upper extremity of the instrument, which is then carried high up into the uterine cavity during the absence of pain. When sufficiently elevated, the free ends are relaxed, and a little gentle manœuvering with the upper end, slips off the loop, and the instrument is then carefully withdrawn. This instrument, though unnecessarily large, seems simple in construction, and easy of application. I have procured one, being determined to make trial of it whenever a case of this unfortunate complication comes under my care.

One of the most formidable maladies which the accoucheur ever encounters, and which may be developed before, during, or after the completion of labor, is eclampsia. My object in referring to this grave affection at this time, is to call your attention to the views relative to its pathology and treatment entertained by the faculty in Vienna. It is well known that an albuminous state of the urine ordinarily precedes and accompanies puerperal convulsions. This remark is sustained by the hospital statistics here furnished. Thus, there have occurred during the last two years, under the care of the gentlemen referred to, twenty-six cases, and in every instance albumen was detected in the urine. In nearly all the cases the albumen existed before the convulsions made their appearance. It is of the utmost importance to bear this in mind, and in cases where apprehensions are entertained, either from the patient's present general health, or from convulsions having been developed during previous labors, that they may make their appearance, the urine should immediately be subjected to an examination; and if, upon the application of heat or nitric acid albuminaria be detected, the practitioner should increase his vigilance during the approaching confinement, and by timely attention he may be able to prevent their development.

Dr. Braun states another peculiarity to be observed in the urine of women who suffer from convulsions. This is a microscopic indication which he thinks is uniformly present, consisting of small flakes or exfoliations of the epithelial membrane lining the *tubuli uriniferi* of the kidneys. This induces me to remark that the microscope is much more extensively used in Europe as an auxiliary means of diagnosis, than in most of the large cities in America. Whether it will prove of value in the diagnosis of the disease under consideration, or not, is yet to be determined, but it is now well established that in ascertaining the nature of many morbid secretions it is, in the hands of those accustomed to its use, an essential aid, and should not be dispensed with. The microscopic appearances referred to as present in eclampsia, seem to consist of minute epithelial scales from the lining membrane of the internal structure of the kidney, agglomerated by means of the mucus. It may be worthy of remark, as showing the value of these signs, that in five cases of "epilepsy habitualis" occurring during labor in the same space of time and in the same wards, the utmost care did not enable Dr. B. to detect either albumen or the microscopic appearances above described. But permit me to give you a little more in detail the history of the twenty-six cases of convulsions which have occurred during the last two years, and of which a careful record has been kept by Dr. Braun. This is the more important in order that we may judge of the treatment pursued, differing, as it essentially does, from the course heretofore ordinarily pursued in England and America. Fourteen of the twenty-six cases occurred after delivery, of which ten recovered, and four died. Several of these, and particularly two of the fatal cases, were delivered in the city, and subsequently, after having had several paroxysms, brought to the hospital. In five cases which were attacked during labor, and in which the forceps were applied, both mothers and children were saved. In four cases occurring before the full period of utero gestation, premature labor was brought on, the children turned, and all safely delivered, saving the children. These are among those already referred to as having labor excited by the india rubber bag, and in all active pains came on within three hours after resorting to the instrûment. In one case the mother died of œdema of the lungs. The cæsarean section was made in this case after her death, but the child was lost. In another there was pelvic deformity, rendering it necessary to excite labor before the complete development of the fœtus. After two days of severe labor, the child being ascertained dead, perforation was resorted to, but the mother died, and a post mortem examination showed the lungs in an œdematous state. The last, entered, after fifteen paroxysms, in an unconscious condition, pulse 140, the mouth of the uterus closed, and strength much exhausted. In one hour the mouth of the uterus dilated, under the influence of the bladder or bag of india rubber, when the child was extracted with the forceps. The mother and child were, however, both lost, and an autopsic examination discovered *apoplectic effusion* into the cavities of the brain. This case, it will be remembered, is the only instance in which blood was found effused, not only in the seven fatal cases just described, but, as I subsequently learned, in those also which had proved fatal during the two previous years.

It is held by Dr. Braun, in common with most of the accoucheurs of this city, that eclampsia is the result of a serous condition of the circulating fluid' a want of fibrin in the blood, which can only be relieved by improving the hæmatosis. He regards bleeding, therefore, not only as useless, but as absolutely increasing the difficulty by impoverishing still more the sanguiferous fluid. He maintains that in the single instance in which blood was found in the cavities of the brain during the last four years, bleeding was inadmissible, in consequence of the state of the pulse and strength.

Again, he ordinarily gives morphine, or some other anodyne, and gives it the more freely if the convulsions come on after delivery, when there can be no danger of retarding the expulsion of the child by its use. The medium dose is 1-16 gr. morphine repeated every quarter to half hour, until the patient becomes quiet, which result is ordinarily obtained by giving from three to six doses, or the half to one gr. of morphine. He does not find the cerebral congestion increased by using opiates, and bleeding, although it might relieve the symptoms for the moment, he deems positively injurious. He prefers small doses of tart antimony if any antiphlogistic remedy be resorted to, and makes cold applications to the head, which he deems much more efficient as a preventive of apoplexy than bleeding.

The statistics of the Vienna lying in-hospital, show that the frequency of the convulsions is not increased by exciting premature labor. In no instance when the female has been attacked before the conclusion of the full term of utero gestation have the paroxysms been rendered more frequent by the use of the "vaginal extensor," although dilation of the uterine orifice and vigorous contractions were in every instance developed. Whenever there were any indications of great cerebral congestion, when the coma was very profound, the cold douche was resorted to, and cold applied continuously to the head. The after treatment consists in quietude, *anodynes*, with slight perspiratives such as Dover's powder, warm nutritious drinks, and a more liberal diet than is ordinarily allowed after parturition. In all cases the albumen disappears in the urine during the eight or ten days which succeed labor without resorting to active medication.

In a subsequent conversation with Dr. Arneth, a distinguished practitioner, who had been the accoucheur in attendance upon the lying-in-hospital during the two years which preceded the record just referred to, I learned that his observations relative to the condition of the patients who suffered from eclampsia, and the treatment which he found most successful, corresponded very nearly with the views just given. During his attendance for that period, 6600 cases of labor came under his individual charge, and a careful record was kept of the whole number. His statistics show four deaths in thirteen cases of convulsions in the mother, and the loss of six children, either at the time or during the nine succeeding days. Dr. A. assures me, that although all fatal cases were examined, in no instance of death after convulsions was there effusion into the cerebral cavities. On the contrary, there was uniformly found an *exsanguine* or *anemic* state of the cerebral mass.

During the early part of his professional life he had resorted to free venesections, but he is now thoroughly convinced that they are neither necessary nor useful. He coincides with Profs. Klien and Braun in the use of opiates, and cold, and in all cases where he detects albuminaria, he gives chloroform as a prophylactic. He believes it allays the nervous irritability, and, when given in moderation, can do no harm, whilst he is convinced he has seen it act favorably in cases of threatened convulsions.

Having myself resorted to the use of chloroform in several cases during the existence of eclampsia, with marked benefit, I am prepared to expect the very best results from its administration under similar circumstances. Some of the cases (one of twins with mal-presentation) have been reported in your excellent Journal, in which the happiest results attended its use. There can be little doubt that there would be much more danger of cerebral congestion (the great danger heretofore apprehended) by the continuance of violent convulsions, than from the calm produced by chloroform, although it may assimilate the coma of effusion somewhat in its immediate effects. Having recently seen chloroform very freely used by Prof. Simpson, of Edinburgh, and seen also many other cases in which it had previously been repeatedly administered in the same subject with apparent benefit, my apprehensions of danger from its inhalation are greatly diminished. Believing, therefore, that chloroform may be resorted to without increasing, in any considerable degree, the cerebral determination, and that it is a most powerful agent in overcoming the terrible spasmodic action of the muscles in cases of puerperal convulsions, I cannot but hope future experience will prove it to be an useful remedy in this fearful disease. Whether the doctrine of the Vienna school in relation to the pathology and treatment of this disease will be sustained by future observers, time alone will determine. It is certain that in many diseases which twenty-five years since were treated by the free use of the lancet and purgatives, are now, without hesitation, given quinine, wine, and generous diet. It is highly probable that the same amelioration in the treatment of this disease will be found best adapted, at least, to a proportion of cases, instead of subjecting all, without discrimination,* to copious venesections.

There is in this admirable institution, a ward especially appropriated to the reception of females suffering from various *post partum* affections. Some

---

* A medical relative, Dr. Edward Flint, of Massachusetts, informed the editor, several years ago, that he had long been accustomed to rely upon morphia in treating cases of puerperal convulsions, having found, in his experience, which had been considerable, this plan of management eminently successful. — EDITOR BUFFALO MED. JOUR.

of the cases now in the hospital are rare, and serve to illustrate the great variety of diseases to which the parturient female is exposed. The first which claimed attention was one of pelvic abcess. The woman entered thirty days previously with a a purulent discharge from the vagina. Upon a careful examination fluctuation was perceived externally, and a free incision made between the glutei muscles, after which the vaginal orifice of the abcess soon closed. Next, Dr. Chiari, the gentlemen in charge of these wards, showed me a female in whom severe labor had been followed by inflammation and suppuration in the tract of the psoas muscle. The abscess was now discharging freely through an external opening, although the amount of purulent matter secreted diminished daily, and the general health of the patient was improving. He then called my attention to an abscess with an external opening in the anterior abdominal walls. The woman had been delivered three months before, and some weeks after labor, was attacked with local or circumscribed peritoneal inflammation, which resulted in abscess.* Dr. C. assures me that instances of this kind are by no means infrequent. They are often the result of imprudence or exposure on the part of the females after they have been discharged. Thus the maternity sends them out, if in good condition, on the ninth or tenth day after labor. Being accustomed to active exertion they resume their duties without sufficient care, being often exposed to cold and wet. Sooner or later a proportion of them return with the various diseases incident to want of care in the parturent state, and among the number he finds many instances of abscess or inflammation of a local character, which often results in suppuration and abscess in the tissues and

---

* Several other cases of a similar character were also in the hospital at this time. One in which a chronic form of circumscribed peritoneal inflammation has produced a firm tumor or mass in the right iliac region, which is doubtless composed of intestines, the right lateral ligament, ovary and uterus itself, firmly agglutinated together. On making a vaginal examination, the uterus is found to be considerably elevated and firmly attached to this mass in the ovarian region. Indeed, the tumor might very naturally, without a careful examination, and remembering the history of the case, be mistaken for ovarian disease. Another patient had extra-peritoneal abscess, which, like those already described, had succeeded some weeks after delivery. And here, again, was a patient with post partum abscess of the abductor muscles of the thigh. The suppuration and burrowing of the pus in this instance, was very extensive, the sinus received a probe two and a half inches upon the anterior part of the thigh, and there is another opening upon the dorsum of the ilium which doubtless gives exit to pus from the same source, although no direct connection can be traced. These cases serve to show the liability to these purulent collections in the lower abdominal and pelvic regions after childbirth, and that there is not necessarily any fault which can attach to the accoucheur.

organs in the neighborhood of the uterus. His treatment, it may be remarked, is based upon general principles adapted to the puerperal state, being careful to make an early and free opening for the escape of the pus. He not infrequently finds the pus deep-seated, and difficult to reach, but deems it of the first importance that it should not be permitted to rupture internally when it can be prevented. When the disease involves the peritoneum and occasions intestinal agglutinations, he resorts to mercurial frictions to the part, being cautious not to carry the mercurialization so far as to produce salivation. He discontinues the mineral entirely, or greatly diminishes the quantity applied, the moment the gums are slightly pricked. The diet is regulated, but nutriment freely allowed, in the cases of protracted abscess, and quietude ordinarily enjoined. Recovery is generally very slowly effected, but the patient seldom dies.

Among the accidents which sometimes result from severe labor, is hernia between the fibres of the recti muscles. A beautiful specimen of this kind was shown me by Dr. Chiari. The tumor, which is on the right side of the mesial line, appeared suddenly, some years since, during the presence of a very severe and protracted labor-pain. It is large and requires support, nevertheless, the woman has, since its existence, been three times pregnant. She was twice delivered at full time, and once aborted without difficulty, or perceptible increase of the hernial tumor.

The number of cases of carcinoma uteri to be found in this institution, is very remarkable. Dr. Chiari informs me that they are of very frequent occurrence in the wards of which he has charge, one in six of all those who enter these wards being affected with that disease. The first indication of its existence which comes to the knowledge of the practitioner, is a sanguineous discharge from the vagina. The woman finds herself loosing blood, applies for remedies, and if it occur during the inter-menstrual period, in ninety to one-hundred cases carcinoma can be detected. This seems almost incredible, but the large number of cases present leaves no room for doubting the correctness of the statement of this accurate observer. The diagnosis of cancer being established, little more is ordinarily attempted than to make the patient comfortable. The victims of this disease seldom enter the hospital until after the proper time (if there be any suitable period for this operation) for excision is passed, and it is seldom or never resorted to in this great charity. Occasionally caustics, such as nitrate of silver, nitrate of mercury, nitric acid, &c., are applied to the diseased surfaces for the purpose of temporarily checking its progress, or arresting the hæmorrhage when inordinate. The chlorate of zinc in solution, is freely injected into the vagina to correct the unpleasant odor always manifest in the advanced stages of this malady. From the entire absence of bad smells in wards containing six or eight patients, in some of whom the uterus is nearly destroyed by ulceration, I am convinced that the fœtor is much more effectually corrected here than in the wards of the English hospitals, where a single case made the atmosphere of a whole ward almost insupportable. It may not be unworthy of remark, also, that in addition to the free use of those anodynes and palliatives ordinarily resorted to, they often administer *conine*, the alkaloid of conium. It is given dissolved in alcohol, in the proportion one grain of conine to one drachm of alcohol, in doses of three or four drops two or three times daily. It should be used with great caution, as one eighth of one grain is said to be poisonous.

With Dr. Fermer I visited the wards of Prof. Sigmund, deservedly celebrated for the treatment of venereal diseases, the Prof. having been for sometime absent from the city. I found them applying a concentrated alcoholic solution of corrosive sublimate to the mucous placques and other analagous morbid growths. It is here thought preferable to the solutions of nitrate of silver, or iodine, ordinarily used for the same purpose in the Parisian hospitals. They had recently, and so far as already tested with satisfactory results, resorted to a preparation of chromic acid. To one part of the acid were added eight of distilled water, or similar proportions, and the parts pointed with the solution in the same manner as when iodem is applied. It is thought that the placques disappear more rapidly from the application of the chromic acid than from the use of the mercurial solution which had previously been the favorite remedy, and is perhaps, even at this moment, more used than the former.

In the constitutional treatment of secondary syphilis there is nothing new worthy of remark. They rely mainly upon the judicious use of the iodides of mercury and potass. The bitter teas or infusions so much in vogue in America and England, within the last few years, are here seldom administered. In the treatment of gonorrhœa I am assured that they rely principally upon topical remedies. Astringent injections into the urethra are freely used, and they are not aware of any instances in which strictures have been the result, unless it be from throwing up a concentrated solution of nitrate of silver when great inflammatory action was present. The sulphates and acetates of lead and zinc, with solutions of tannic acid in such strength as the condition of the membrane will warrant, are chiefly resorted to. Great confidence is also placed in the application of cold water to the penis during the inflammatory stage. Dr. Fremer does not recollect an instance of the troublesome erections, or chordee, supervening whilst the patient was under the cold water treatment. Meantime the patient is restricted to a light diet, quietude is enjoined, and saline aperients moderately given, but seldom, or never, do they administer copaiba or cubebes. In gleety or chronic urethral discharges, nitrate of silver injections are thrown up daily until the action of the membrane is modified, when mild astringents will ordinarily perfect the cure. Prof. Schuh treats stricture of the urethra by forcible dilitation, thus, as he maintains, overcoming, in a few days, a contraction which, under the old method of treatment, it often required months to dilate. He introduces a small metallic bougie, which is divided, longitudinally, in the centre, almost to the point, and held together by a screw running transversely at the outer extremity. When fairly engaged in the stricture he turns the screw which separates the sides of the bougie, and thus dilates the stricture with great rapidity. Several instances are reported as having been operated upon as above described, with satisfactory results.

Although your patience may long since have been exhausted, I cannot close this communication without asking your indulgence whilst some slight reference is made to the ophthalmic wards in this great hospital. Vienna has long enjoyed a high reputation in this department, having produced many illustrious names, and furnished works which have been made text-books throughout the world. She still ranks among her surgeons some of the first oculists of the day, and her museums are especially rich in every thing pertaining to the pathology of the eye. Being introduced to the celebrated Rosas, by my friend Dr. Seidl, an occulist of considerable eminence, whose acquaintance I had made in Paris, my attention was chiefly confined to his operations and lectures. As peculiar to the Vienna school at this time, it may be remarked, that, so far as my observations enable me to judge, cataract is ordinarily depressed, or reclined and divided, rather than extracted. The French, on the contrary, usually extract. Dr. Rosas seems careful to rupture the capsule and break up the lens as much as possible, without doing injury to the surrounding tissues. For this purpose he uses a long needle with the shoulders very broad, as compared with those to be found in the hands of American surgeons, The operation is repeated, if necessary, in order to entirely get rid of the obstruction to the rays of light. I saw Dr. R. operate upon an eye which had previously been subjected to the needle, and on which the lens itself had nearly disappeared, but the thickened opaque anterior capsule still remained occupying the pupillary axis. He divided the membrane freely, and at the same time pushed it below the pupil. It may be remarked, in passing, that Dr. Rosas is ambidextrous, using the left equally well with the right hand, which is exceedingly convenient in operations upon the eye. He is now sixty years old, and obliged to wear spectacles when operating. One thing struck me as remarkable, which was, that in all these operations for artificial pupil, cataract, &c., &c., they did not resort to belladonna previously. Upon inquiry, I was however told that it was occasionally applied.

Yours truly, James P. White.

